# Calcitonin inhibits intervertebral disc degeneration by regulating protein kinase C

**DOI:** 10.1111/jcmm.15496

**Published:** 2020-06-21

**Authors:** Jun Ge, Xiaoqiang Cheng, Qi Yan, Cenhao Wu, Yingjie Wang, Hao Yu, Huilin Yang, Feng Zhou, Jun Zou

**Affiliations:** ^1^ Department of Orthopaedic Surgery The First Affiliated Hospital of Soochow University Suzhou China

**Keywords:** calcitonin, intervertebral disc degeneration, protein kinase C, rat caudal model

## Abstract

Intervertebral disc degeneration (IVDD) is the most critical factor that causes low back pain. Molecular biotherapy is a fundamental strategy for IVDD treatment. Calcitonin can promote the proliferation of chondrocytes, stimulate the synthesis of matrix and prevent cartilage degeneration. However, its effect and the underlying mechanism for IVDD have not been fully revealed. Chondrogenic specific matrix components’ mRNA expression of nucleus pulposus cell (NPC) was determined by qPCR. Protein expression of NPC matrix components and protein kinase C was determined by Western blotting. A rat caudal intervertebral disc degeneration model was established and tested for calcitonin in vivo. IL‐1 induced NPC change via decreasing protein kinase C (PKC)‐ε phosphorylation, while increasing PKC‐δ phosphorylation. Calcitonin treatment could prevent or reverse IL‐1‐induced cellular change on PKC signalling associated with degeneration. The positive effect of calcitonin on IVDD in vivo was verified on a rat caudal model. In summary, this study, for the first time, elucidated the important role of calcitonin in the regulation of matrix components in the nucleus of the intervertebral disc. Calcitonin can delay degeneration of the intervertebral disc nucleus by activating the PKC‐ε pathway and inhibiting the PKC‐δ pathway.

## 1 INTRODUCTION

Low back pain (LBP) has become a significant public health concern worldwide as the ageing population increases globally.^1^ Since 1990, the number of people living with LBP‐associated disabilities has increased by over 50%, especially in low‐income countries,^2^ and the high incidence rate of LBP and LBP‐induced disability has been drawing considerable attention worldwide. Intervertebral disc degeneration (IVDD) is the most critical factor that causes LBP.^3^ The current therapeutic approaches for IVDD include bed rest, administration of non‐steroid anti‐inflammatory drugs (NSAIDs), lumbar discectomy and interbody fusion. Contemporary treatment only improves the clinical symptoms, but cannot delay or prevent the degeneration of the intervertebral disc. Although surgical intervention produces a promising short‐term outcome, the recurrence rate of LBP is usually high, and the degeneration of adjacent discs is accelerated after surgery.^4^ Unlike the conservative therapeutic approaches, molecular biotherapy aims at preventing or reversing the degeneration of the extracellular matrix of the intervertebral disc, and controlling IVDD development.^5^ Thus, this fundamental strategy has drawn essential attention in IVDD treatment.

The causes of IVDD mainly include genetic factors, age, loss of proteoglycan, water and type II collagen,^5^ as well as insufficient transport of metabolites.^6^ Nucleus pulposus (NP) is an inner gel‐like tissue in the intervertebral discs. NP originates from the notochord nucleus pulposus, which is necessary for proteoglycans and water reservation in NP.^7^, ^8^ Insufficient nutrient supply to the intervertebral disc induced by the degeneration of cartilage endplate is the primary cause of IVDD.

It has been clarified that there are no significant differences of calcitonin and calcitonin receptor expression between normal and degenerated NPC.^9^ Changes in endogenous calcitonin may not be a key factor in the degeneration of NPCs. Moreover, a large number of clinical and fundamental studies have shown that calcitonin can promote the proliferation of chondrocytes, stimulate the synthesis of type II collagen and proteoglycans, and prevent cartilage degeneration.^10^, ^11^, ^12^, ^13^, ^14^ In support of this theory, some recent studies have reported the role of exogenous calcitonin in blocking IVDD degeneration.^15^, ^16^, ^17^ The former studies all focused on the ovariectomized osteoporotic animals. The osteogenic effect of calcitonin could preserve the microarchitecture and biomechanical properties of adjacent vertebrae, and finally prevent IVDD.^15^, ^16^, ^17^ Moreover, both Tian et al and Luo et al found that calcitonin could protect ovariectomized rats from IVDD by modifying extracellular matrix metabolism of the intervertebral discs.^15^, ^16^ Hence, calcitonin must have a direct protective effect on NPCs. However, none of these studies have investigated the mechanism by which calcitonin blocks IVDD. Therefore, it is necessary to elucidate the molecular biology of IVDD for the development of the targeted therapy for this disease.

In recent years, a large number of studies focused on the signalling pathways related to IVDD.^18^, ^19^ Recent studies have shown that the protein kinase C (PKC) pathway is involved in regulating the synthesis of type II collagen and proteoglycans in nucleus pulposus cells (NPCs). It also plays an essential role in the synthesis and degradation of the extracellular matrix (ECM). Taking into account the characteristics of chondroid of NPCs and the critical role of calcitonin in chondrocyte matrix synthesis, we hypothesized that calcitonin might play a role in delaying IVDD through the PKC pathway. Thus, to find an effective method to prevent disc degeneration, we aimed to further elucidate the underlying mechanisms and the role of calcitonin in IVDD through a series of in vitro and in vivo experiments.

## 2 MATERIALS AND METHODS

IL‐1β (R&D Systems, Minneapolis, MN, USA) was dissolved in sterile phosphate‐buffered saline (PBS, pH 7.4, 0.01 M) containing 0.1% BSA to obtain a concentration of 25 μg/mL. It was then diluted to different concentrations with culture medium prior to cell exposure. Similarly, calcitonin (Sigma) was diluted to different concentrations with culture medium before cell treatment. All the experimental procedures were approved by the Ethics Committee of the First Affiliated Hospital of Soochow University and were carried out in strict accordance with the Declaration of Helsinki (1964) and the Laboratory Animal Guidelines for Ethical Review of Animal Welfare (GB/T 35892‐2018, China).

### 2.1 Isolation and culture of primary nucleus pulposus cells

The method is as follows: a male clean Sprague Dawley (SD) rat (GB 14922‐1994) weighing 225‐250 g was killed and then disinfected in 75% alcohol for 15 minutes. The root of the tail (including the caudal vertebral disc tissue) was cut under sterile conditions and peeled to full exposure of the tail intervertebral disc. It was subsequently transferred to a glass plate containing 2 × penicillin‐streptomycin in PBS for 5 minutes. The intervertebral disc fibre ring was cut using a sterile sharp surgical blade, and the NP tissue was completely removed and placed in PBS containing 100 × penicillin‐streptomycin. The tissue was cut into 1 mm^3^ pieces and moved to individual sterile centrifuge tubes. Type II collagenase solution (0.2%) was added to the tissue at a 5:1 volume ratio, followed by incubation in a water bath at 37°C for 2 hours with gentle shaking every 20 minutes to digest the tissue. The digestion was neutralized by the addition of an equal volume of DMEM/F12 complete culture medium, followed by centrifugation at 1000 rpm for 5‐8 minutes. The supernatant was aspirated, and the cells were resuspended in fresh DMEM/F12 complete culture medium and transferred to a culture flask. The cells were cultured in a saturated humidity incubator with 5% CO_2_ at a constant temperature of 37°C. All the experimental procedures were performed at the First Affiliated Hospital of Soochow University. Ten intervertebral discs from two rats are necessary for the isolation primary nucleus pulposus cells (NPCs) at a time. New primary NPCs were isolated and subcultured to the 3rd generation before each experiment (including repeated experiments).

### 2.2 Cell culture and treatment

NPCs were cultured in F12 medium supplemented with 10% (v/v) foetal bovine serum, 100 units/mL of penicillin, 100 mg/mL streptomycin at 37°C and 5% CO_2_. For the following quantitative real‐time PCR (RT‐qPCR) and Western blotting assays, the cells were seeded into a 10 cm dish before treating them with IL‐1β, when the cell confluency reached 70%. Solutions of different concentrations of IL‐1β (0.1‐100 ng/mL) and calcitonin (0.1‐300 ng/mL, Calcitonin Salmon, MedChemExpress, Cat. No.: HY‐P0090) were added to the cells, and they were cultured for 24 hours.

### 2.3 RT‐qPCR

Total RNA was extracted from the cells that underwent different treatments using the RNeasy kit (QIAGEN, Germany). The cDNA was synthesized using RevertAid First Strand cDNA Synthesis Kit (Thermo Scientific, CA, USA), according to the protocol provided by the manufacturer. RT‐qPCR was performed with an ABI 7500 sequence detection system (Applied Biosystems, Foster City, CA) and Power SYBR Green PCR Master Mix (Applied Biosystems). Each target probe was amplified in a separate 96‐well plate. Sequences of target genes could be found in Table 1. All samples were incubated at 95°C for 10 minutes. They were then cycled at 95 ºC for 15 seconds, 60°C for 1 minutes for 40 cycles. The results were evaluated using the SDS software 7300 (Applied Biosystems) and Microsoft Excel. For each sample, the relative amount of the target mRNA was determined and normalized to GAPDH.^20^ The data were presented as the fold change using the formula 2^−ΔΔCT^, as recommended by the manufacturer. All experiments were repeated three times. All the gene expression level in each parallel sample is compared and normalized with the control group. Relative gene expression levels were finally achieved.

**Table 1:** Names and sequences of target genes and a house‐keeping reference GAPDH gene

Primer	Sequence
Collagen II‐F	TGCTGCCCAGATGGCTGGAGGA
Collagen II‐R	TGCCTTGAAATCCTTGAGGCCC
Aggrecan‐F	CTACCGCTGCGAGGTGATG
Aggrecan‐R	AGTCGAGGGTGTAGCGTGTAGAG
CollagenX‐F	TGCTGCCACAAATACCCTTT
Collagen X‐R	GTGGACCAGGAGTACCTTGC
GAPDH‐F	CCCCCAATGTATCCGTTGTG
GAPDH‐R	TAGCCCAGGATGCCCTTTAGT

### 2.4 Western blotting

Total protein was extracted from the cells treated with different concentrations of IL‐1β and calcitonin using RIPA buffer (50 mmol/L Tris, pH 7.4; 150 mmol/L NaCl; 1% NP‐40; 0.5% sodium deoxycholate). The treated cells were rinsed with cold PBS three times. Next, 200 μL cold RIPA buffer was added into the dish, and the solution was collected and centrifuged at 13 000 *g*, 4°C for 20 minutes. The supernatant was transferred to a new centrifuge tube, and the protein concentration was quantified using BCA Kit (Beyotime, China). After the 5× loading buffer (250 mmol/L Tris‐HCl, pH 6.8; 10% (w/v) sodium dodecyl sulphate (SDS); 0.5% (w/v) black‐pigmented bacteroides; 50% (v/v) glycerinum; 5% (w/v) β‐mercaptoethanol) was added and boiled, the proteins (40 μg) were separated on 10% SDS polyacrylamide gels under reducing conditions and then transferred on polyvinylidene fluoride (PVDF) membranes. After blocking the nonspecific binding sites with 5% low‐fat milk powder in Tris buffer solution (TBS) containing 0.1% Tween‐20, PVDF membranes were incubated with a polyclonal antibody (anti‐Collagen II (Ab34712, 1:2000), anti‐Collagen X (Ab58632, 1:200), anti‐PKC‐ε (Ab124806, 1:1000), anti‐p‐PKC‐ε (Ab63387, 1:1000), anti‐PKC‐δ (Ab182126, 1:1000), Abcam; anti‐aggrecan (13880‐1‐AP, 1:1000), Proteintech; anti‐p‐PKC‐δ (2055, 1:1000), Cell Signaling Technology) in a dilution of 1:1000 in 2.5% low‐fat milk powder overnight at 4°C. Horseradish peroxidase‐conjugated goat anti‐rabbit antibodies (Cell Signaling Technology) diluted 1:10 000 were used as secondary antibodies. Immunoreactive proteins were visualized using a chemiluminescence kit (Thermo) followed by exposure under gel imaging device (LAS4000EPUVmini, Fujifilm). The images were analysed after data collection using ImageJ software. For protein expression, the intensities of the proteins were compared with those of the internal reference proteins. The ratios were then compared with those of the control group, while the phosphorylation level of PKC is the ratio of intensities of p‐PKC and PKC. All experiments were repeated three times.

### 2.5 Establishment of rat caudal IVDD model

A total of 24 clean SD rats (male, weighing 400 ± 20 g, GB 14922‐1994) were provided by the Animal Experimental Center of Soochow University. After magnetic resonance scanning and X‐ray examination to confirm the absence of any congenital malformations or disc degeneration of the caudal vertebrae, the rats were randomly divided into four groups by the digital table method: normal group, degeneration group, calcitonin group and normal saline (NS) group, each with six rats. Preoperative X‐rays were used to position Co 9/10 vertebral gaps. The rats were weighed and then intraperitoneally anaesthetized with ketamine hydrochloride (50 mg/kg) and xylazine hydrochloride (5 mg/kg). The rats were fixed onto the operating table, and 18G injection needles were used to pierce the Co 9/10 vertebral gaps for degeneration group, calcitonin group and normal saline (NS) group. The needle tips were inserted perpendicular to the rat tail until complete penetration to the opposite side, then rotated 360°C and retracted after 30 seconds. After surgery, the rats were allowed to move freely in the cage, with no dietary ban, and were strictly observed for the presence of urinary retention and infection.

One week later, magnetic resonance imaging (MRI) analysis confirmed the successful establishment of the IVDD rat model. The degeneration group was punctured without any drug treatment. According to the previous study, 2 μL is the maximal injection volume for the rat caudal model.^21^ In order to achieve the best therapeutic effect, an injection volume of 2 μL was selected for the following treatment. After the MRI scan, 2 μL of calcitonin (20 µg/mL) or normal saline was injected into the degenerated intervertebral discs of the rats in the calcitonin and NS groups, respectively, using a 26G thick needle.

### 2.6 MRI examination

All the rats were intraperitoneally anaesthetized with 10% chloral hydrate (3.5 mL/kg) and then scanned with an MRI on the 2nd and 4th weeks after the injection. Intervertebral disc signals were obtained on 1.5T magnetic resonance (MR) scanner (Philips Eclipse), using the following parameters of the T2‐weighted sagittal plane: TR/TE: 3500/102 ms, FOV: 15.0, thickness: 3 mm, interval: 0 mm. The degree of disc degeneration was assessed by signal intensity on T2‐weight image (T2WI) of intervertebral disc and graded with a five‐grade Pfirrmann system^22^ (Grade I: The structure of the disc is homogeneous, with a bright hyperintense white signal intensity and a normal disc height. Grade II: The structure of the disc is inhomogeneous, with a hyperintense white signal. The distinction between nucleus and annulus is clear, and the disc height is normal, with or without horizontal grey bands. Grade III: The structure of the disc is inhomogeneous, with an intermediate grey signal intensity. The distinction between nucleus and annulus is unclear, and the disc height is normal or slightly decreased. Grade IV: The structure of the disc is inhomogeneous, with an hypointense dark grey signal intensity. The distinction between nucleus and annulus is lost, and the disc height is normal or moderately decreased. Grade V: The structure of the disc is inhomogeneous, with a hypointense black signal intensity. The distinction between nucleus and annulus is lost, and the disc space is collapsed.).

### 2.7 Histological examination

After the imaging examination, the rats were killed and the Co 9/10 discs were extracted entirely and fixed with 10% neutral formaldehyde at room temperature for 24 hours. The discs were decalcified in 10% EDTA for 2 weeks and then sliced into five horizontal sections of 4 μm thickness. Slices were dewaxed in xylene twice for 5 minutes, dehydrated in graded ethanol (start from 75%, 1 minutes; 80%, 1 minutes; 95%, 1 minutes; 100%, 2 minutes), and stained with haematoxylin for 5 minutes and eosin for 2 minutes (H&E staining) or stained with Fast Green for 5 minutes and Safranin O for 5 minutes (Safranin O Fast Green staining). The morphology of the intervertebral disc was observed and scored under a light microscope, based on Masuda Standard, according to Table 2.^23^, ^24^, ^25^ The method of killing was approved by the Ethics Committee of the First Affiliated Hospital of Soochow University and was performed in strict accordance with the Declaration of Helsinki (1964) and the Laboratory Animal Guidelines for Ethical Review of Animal Welfare (GB/T 35892‐2018).

**Table 2:** Grading for morphology

		Morphology Change Under Optical Microscope	Grade
Ⅰ	Annulus Fibrosus (AF)	Normal texture and free of damage and distortion	1
		The damaged and distortion area is less than 30%	2
		The damaged and distortion area is more than 30%	3
Ⅱ	Boundary between AF and NP	Normal	1
		Micro disrupted	2
		Medium or severe disrupted	3
Ⅲ	NP Cells	Normal cells with large amounts of vacuoles	1
		Cells and vacuoles decreased slightly	2
		Cells decreased moderately or severely without vacuoles	3
Ⅳ	NP Matrix	Normal gel appearance	1
		Slightly congealed	2
		Moderate or severe condensation	3

### 2.8 Immunohistochemical staining

The expression of collagen II, collagen X and PKC was detected using immunohistochemistry. The slices were dewaxed in xylene, dehydrated in graded ethanol and incubated in 3% H_2_O_2_ at 37°C for 10 minutes. Next, they were washed in PBS for 5 minutes thrice, boiled in 0.01 M citric acid buffer for antigen retrieval (95°C, 15‐20 minutes) and blocked in goat serum for 10 minutes at 37°C. Slices were then incubated with primary antibody (anti‐Collagen II (1:200), anti‐Collagen X (1:50), anti‐PKC‐ε (1:50) and anti‐PKC‐δ (1:1000), Abcam, USA) at 4°C overnight and with biotin‐labelled secondary antibody (Bioworld) for 30 minutes at 37°C. Slices were counterstained with haematoxylin and observed under a light microscope.

### 2.9 Statistical analysis

All quantitative data are presented as mean ± SD Statistical analyses were performed using one‐way ANOVA. For non‐parametric data, the Kruskal‐Wallis test was performed. Differences with values of *P* < .05 were considered statistically significant.

## 3 RESULTS

### 3.1 IL‐1 treatment induces NPC cellular change

**Figure 1:**
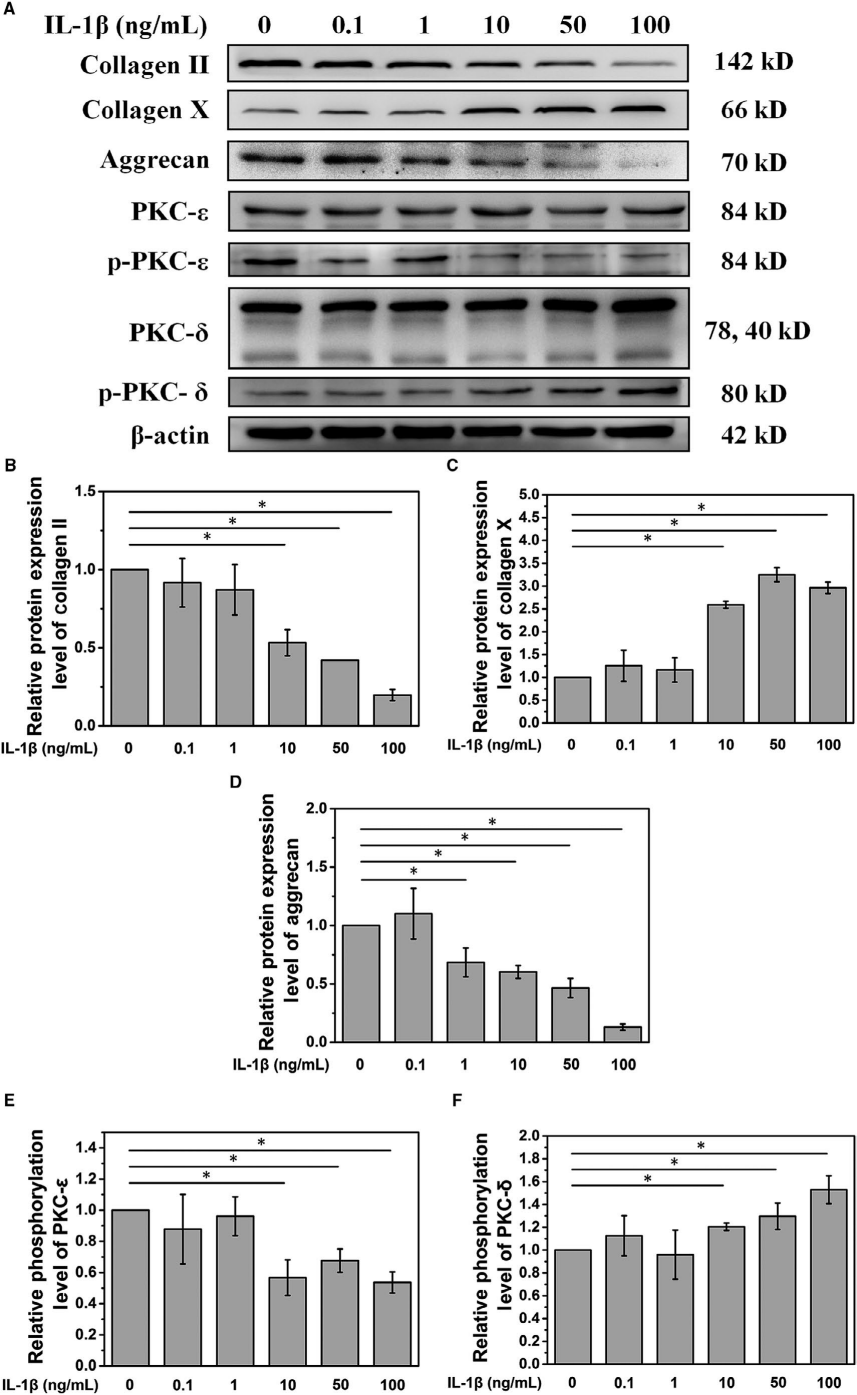
A. IL‐1 treatment decreases PKC‐ε phosphorylation and increases PKC‐δ phosphorylation. B, C and D. Quantitative analysis of the Western blot showed that at a concentration of over 10 ng/mL, IL‐1 could significantly affect the expression of type II collagen, aggrecan and type X collagen. E and F. Quantitative analysis of the Western blot showed that a concentration of IL‐1 over 10 ng/mL could significantly affect PKC signalling (*P < .05)

Our data showed that IL‐1 induces the cellular change of NPCs. Moreover, IL‐1 decreased the protein expression of type II collagen and aggrecan while increasing that of type X collagen in a dose‐dependent manner (Figure 1A). At a concentration of over 10 ng/mL, IL‐1 could significantly affect the expression of type II collagen, aggrecan and type X collagen (*P* < .05, Figure 1B‐D). Thus, a 10 ng/mL concentration of IL‐1 was selected for further investigation.

### 3.2 IL‐1 treatment affects PKC signalling

Analysis of PKC signals showed that IL‐1 decreased the phosphorylation level of PKC‐ε and increased the phosphorylation of PKC‐δ in NPCs (Figure 1A). Further quantification analysis showed that a concentration of IL‐1 over 10 ng/mL could significantly affect PKC signalling (*P* < .05, Figure 1E and F).

### 3.3 *Calcitonin delays IL‐1‐induced NPC cellular change* in vitro

**Figure 2:**
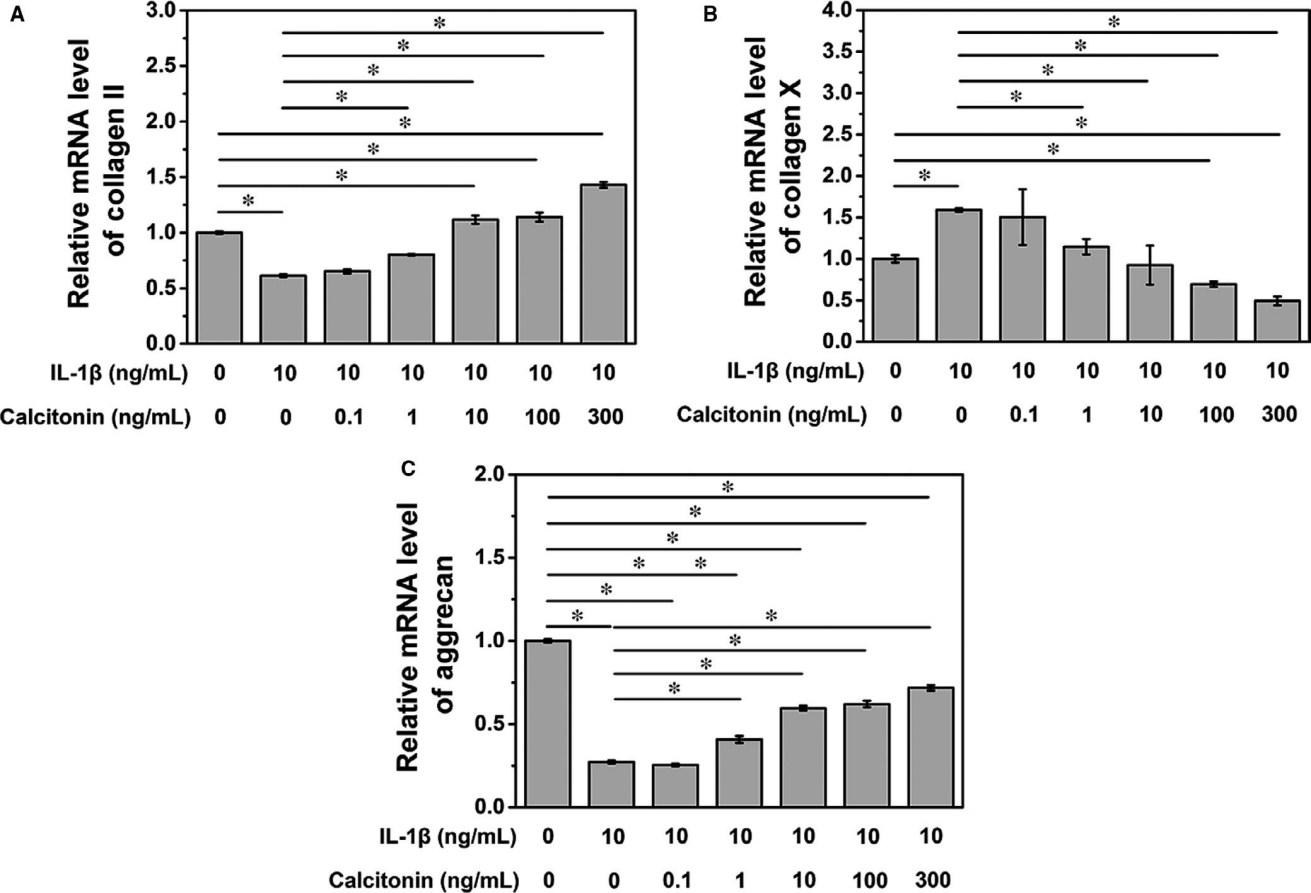
A. IL‐10 treatment could decrease the inhibition effects of IL‐1 on collagen II mRNA expression (*P < .05). B. IL‐1 increases the mRNA expression of collagen X, while IL‐10 treatment could reduce collagen X mRNA expression in a dose‐dependent manner (*P < .05). C. IL‐10 treatment could alleviate the inhibition effects of IL‐1 on aggrecan mRNA expression (*P < .05)

**Figure 3:**
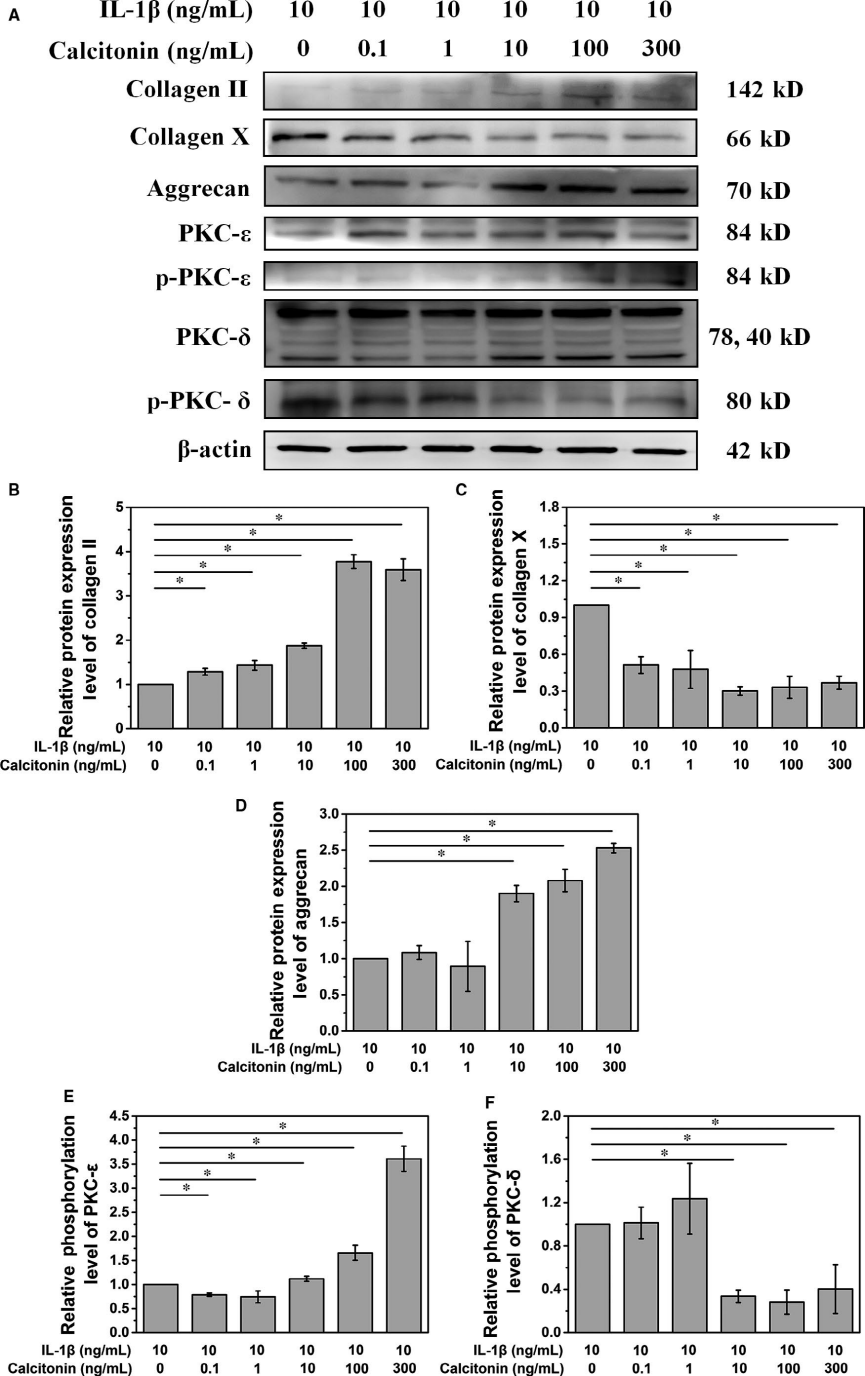
A. IL‐10 treatment stimulates the synthesis of the NPC matrix via activating the PKC‐ε pathway and inhibiting the PKC‐δ pathway. B, C and D. Quantitative analysis of the Western blot showed that a concentration of IL‐10 over 10 ng/mL could significantly affect the synthesis of the NPC matrix (*P < .05). A low concentration of IL‐10 treatment could increase the protein level of collagen II while decreasing that of collagen X (*P < .05). E and F. A concentration of IL‐10 over 10 ng/mL could significantly affect the PKC‐ε pathway and inhibiting the PKC‐δ pathway (*P < .05)

NPCs were pre‐treated with 10 ng/mL of IL‐1 followed by the addition of calcitonin at different doses. Data from PCR analysis showed that calcitonin gradually increased the mRNA levels of type II collagen and aggrecan in a dose‐dependent manner, with significant changes in the levels of type II collagen and aggrecan in NPCs treated with calcitonin (dose > 1 ng/mL, *P* < .05, Figure 2A and C). Similarly, type II collagen and aggrecan protein expression gradually increased in NPCs following calcitonin treatment (Figure 3A, B, and D). In contrast, the expression of type X collagen, an indicator of degenerated NPCs, reduced after calcitonin treatment (Figures 2B and 3C). Collectively, these data suggest that calcitonin can delay IL‐1‐induced cellular change of NPCs.

### 3.4 Calcitonin reverses the cellular change of NPCs via PKC pathway

To further explore the mechanism by which calcitonin delays the degeneration of NPCs, we examined the total expression level and phosphorylation status of PKC. Our results showed that calcitonin increased phosphorylated PKC‐ε but reduced phosphorylated PKC‐δ, which were antagonistic to the effects of IL‐1 treatment (Figure 3 A, E, and F). These data suggest that calcitonin reverses the effects of IL‐1 on these two critical pathways, thereby reversing the cellular change of NPCs.

### 3.5 Calcitonin delays the IVDD in a rat model

First, we established a rat model of IVDD using the acupuncture method, which was divided into the degenerative group, calcitonin group and normal saline (NS) group. In addition, the normal group without puncture was also used in the current study. In the first week, the rats in punctured group were subjected to MRI analysis. After confirming the IVDD, rats were injected with calcitonin and NS in the calcitonin group and NS group, respectively.

**Figure 4:**
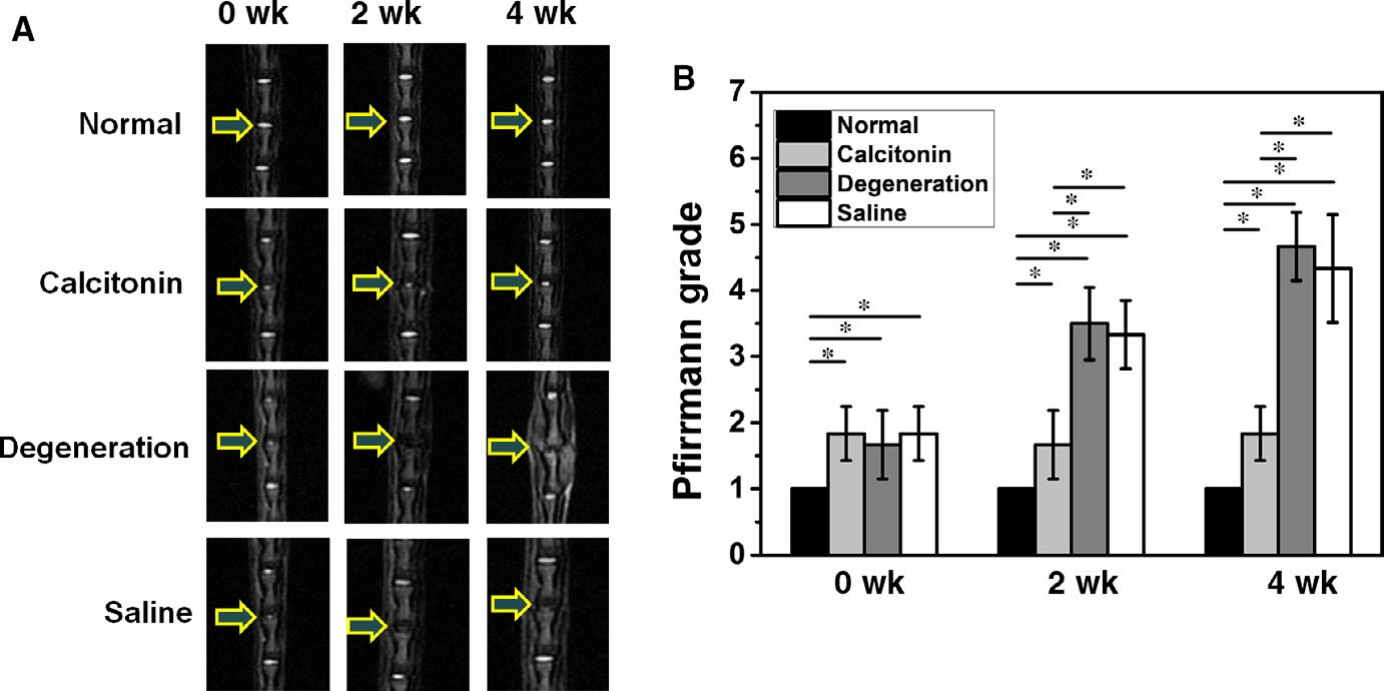
A. MRI scan showed that calcitonin alleviates the rat caudal IVD in the 4th week after injection. B. Pfirrmann score showed that calcitonin alleviates the rat caudal IVD morphologically (*P < .05)

Magnetic resonance imaging results reveal that the intervertebral discs of saline and degeneration groups get gradually darker with increasing time. Besides, the spinal space also becomes narrower. In the calcitonin group, a high signal of intervertebral disc was detected 4 weeks after the injection (Figure 4A). However, compared with the normal group, the signal strength and intervertebral height of calcitonin group was lower. Consequently, the Pfirrmann grade of calcitonin‐treated rats was significantly lower than those of the degeneration and saline groups, but was significantly higher than normal group (Figure 4B).

**Figure 5:**
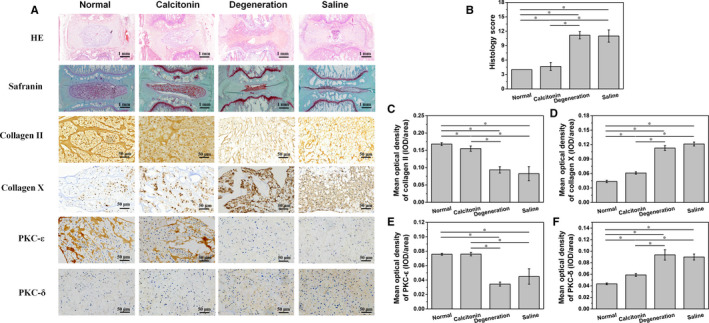
A. Histological analysis of rat caudal IVDD in the 4th week after injection. B. Semi‐quantitative analysis of HE staining showed that calcitonin alleviates the rat caudal IVDD histologically. C. The mean optical density of collagen II in the calcitonin group is higher than in degeneration and saline groups, but lower than normal group (*P < .05). D. The mean optical density of collagen X in the calcitonin group is lower than in degeneration and saline groups, but higher than normal group (*P < .05). E. The mean optical density of PKC‐ε in the calcitonin group is higher than in degeneration and saline groups (*P < .05). F. The mean optical density of PKC‐δ in the calcitonin group is significantly lower than in degeneration and saline groups, but higher than normal group (*P < .05)

In the fourth week after the injection, histological analysis revealed a clear boundary between NP and fibrous ring after treatment with calcitonin (Figure 5A). Additionally, compared to the degeneration and NS groups, the number of chondrocytes in NP increased in the calcitonin group. The annulus fibrosus in the calcitonin group showed a regular ring structure, and the layers were neatly arranged without significant degradation. In contrast, rats in the NS and degeneration groups exhibited significant degeneration in NP; some rats did not display NP. Semi‐qualified analysis showed a better morphology after calcitonin treatment (*P* < .05, Figure 4B). Although calcitonin group showed a satisfying therapeutic effect, quantitative analysis revealed significant differences compared with the normal group, indicating calcitonin can only delay the process of degeneration.

Immunohistochemical analysis showed that type II collagen increased in calcitonin‐treated rats (Figure 5A and C), while immunohistochemical analysis of collagen X showed the opposite result (Figure 5A and D). Besides, calcitonin treatment induced significant up‐regulation of PKC‐ε (Figure 5A and E) and down‐regulation of PKC‐δ (Figure 5A and F) compared to rats in NS and degeneration groups. Similar to histological analysis, there was a significant difference of immunohistochemical optical density between calcitonin and normal group. It suggested that calcitonin might not be able to reverse intervertebral disc degeneration.

## 4 DISCUSSION

In the present study, we explored the relationship between calcitonin and NP in IVDD and investigated the underlying mechanism by which calcitonin delayed IVDD development. Considering the crucial role of calcitonin and PKC pathway in the matrix production of NP, we hypothesized that calcitonin might play a role in delaying the degeneration of IVDD through the PKC pathway. To validate this hypothesis, we studied the effect of IL‐1 on the mRNA and protein expression levels of collagen II/X and aggrecan and examined the phosphorylation state of PKC‐ε and PKC‐δ in IL‐1‐treated NPCs. We found that at a high dose (>10 ng/mL) IL‐1 decreased the mRNA and protein expression of type II collagen and aggrecan, but increased type X collagen mRNA and protein expression. Type II collagen and aggrecan are essential components in the extracellular matrix of NPCs. Studies have shown that type II collagen in NPCs is negatively associated with the severity of IVDD.^26^, ^27^, ^28^ Proteoglycan aggrecan promotes the stress resistance of IVDD through its hydrophilic chondroitin sulphate chains.^29^ Type X collagen is a short‐chain, non‐fibrillar collagen with a network structure that cross‐links with type II collagen, affecting the structure and strength of the intervertebral disc. Type X collagen is often detected in the calcified tissues,^30^, ^31^ revealing a close relationship between type X collagen and calcification. A large amount of type X collagen is detected in chondrocytes in degenerative intervertebral disc.^32^ Thus, type X collagen is critical for IVDD and repair. In summary, the degeneration‐like cellular change of NPCs occurs at a high dose of IL‐1 (>10 ng/mL), which is mediated by the PKC pathway. IL‐1 inhibits the activation of PKC‐ε pathway and promotes the activation of PKC‐δ pathway. These results suggest that IL‐1 induces the cellular change of NPCs through the PKC pathway.

The PKC pathway is critical in cell growth and differentiation. PKC isoforms include three subfamilies: the classical (cPKC), novel (nPKC) and atypical (aPKC) PKCs.^33^ PKC isoforms exhibit different roles in NPCs. As a member of nPKC, PKCδ is an upstream regulator of the MAPK (ERK, JNK, p38) signalling cascades.^34^ Ellman et al^35^ have reported that suppression of the PKCδ pathway can prevent IVDD. Yokoyama et al^18^ have found that c‐Fos, an important factor in the pathogenesis of IVDD degeneration, is inhibited by activation of the PKC pathway. Although PKCδ is highly expressed, it is not able to induce a high expression of c‐Fos. Other studies suggest that the PKC pathway promotes the degradation of β‐catenin and subsequently inhibits Wnt/β‐catenin pathway.^36^ Arai et al^19^ demonstrated that activation of PKC signalling, with a high expression of PKCε and PKCγ, leads to an increase in matrix synthesis and cell proliferation in NPCs, which prevents IVDD. Consistent with these studies, our results showed that IL‐1 inhibited the activation of PKC‐ε but promoted PKC‐δ activation, thereby inducing NPC degeneration. Based on these findings, we further hypothesized that calcitonin might delay the degeneration of NPCs by activating or inhibiting different PKC isoforms. This hypothesis was validated in the following experiments.

Calcitonin has been shown to play a protective role against IVDD.^15^, ^16^, ^17^, ^37^, ^38^ Previous studies mainly focused on the effect of calcitonin on the regulation of calcium and phosphorus.^15^, ^16^, ^37^ Calcitonin regulates the morphological integrity of the cartilage endplate of the vertebral body and affects the biomechanics of IVDD, and subsequently alleviates IVDD. Some studies have also been conducted to investigate the role of matrix components in the annulus fibrosus in the degeneration of the intervertebral disc.^17^ The regulatory function of matrix indicates the interaction between calcitonin and NPCs. However, the role of calcitonin in NPCs and its underlying mechanisms remains unclear. Therefore, this study, for the first time, focused on the relationship between calcitonin and NP of the intervertebral disc. First, we confirmed the role of calcitonin in delaying the degeneration of NPCs. Treatment with calcitonin (<1 ng/mL) significantly increased the mRNA and protein expression of type II collagen and aggrecan in IL‐1‐stimulated NPCs. Moreover, calcitonin reduced the mRNA and protein expression of type X collagen, indicating that calcitonin could delay the IL‐1‐induced NPC degeneration.

Furthermore, calcitonin increased the expression of phosphorylated PKC‐ε, suggesting its activation effect on the PKC‐ε pathway. Meanwhile, calcitonin reduced the levels of phosphorylated PKC‐δ, suggesting its inhibitory effect on the PKC‐δ pathway. These results are in contrast to the role of IL‐1 on PKC‐ε and PKC‐δ pathways, suggesting that calcitonin reverses the effects of IL‐1 on these two critical pathways, thereby delaying the degeneration of NPCs. The role of calcitonin on matrix production in the chondrocytes has been validated previously. Recently, Zhang et al^39^ have found that calcitonin plays a protective role in chondrocytes by down‐regulating MAPK/Wnt/NF‐κB pathway. As isogenous cells of cartilage, calcitonin plays a protective role in the pathogenesis of IVDD by regulating the pathways mentioned above. However, the study by Zhang et al did not directly elucidate the specific mechanism by which calcitonin inhibits MAPK/Wnt/NF‐κB pathway. In order to further explore the protective role of calcitonin, it was necessary to detect calcitonin‐regulated upstream pathways. PKC is critical for the regulation of MAPK and Wnt pathways. We found that calcitonin also regulates the PKC signalling pathway.^40^ Considering the critical role of PKC in matrix production in intervertebral disc, we studied the relationship between calcitonin and PKC pathway. Our results showed that calcitonin can activate PKC‐ε and inhibit PKC‐δ, thereby exerting its protective role for IVDD.

Our hypothesis that calcitonin maintains the normal disc structure was further validated by in vivo assays. Considering the availability of antibodies, cost, similarity to human biochemical components, and reliability, rat IVDD model has been widely used.^24^ There are many ways to establish a rat IVDD degeneration model, for example, biomechanical stress model,^41^ spinal instability model,^42^ retroperitoneal drilling model,^43^ chemical injury model^44^ and others. Among them, the puncture model for caudal IVDD is a very convenient way and has been meanwhile accepted by many laboratories. Caudal IVDD of rats is more convenient to be located and operated compared to the lumbar IVDD. Several studies have applied the rat caudal IVDD degeneration model for morphological, biological and molecular research.^45^, ^46^, ^47^, ^48^ Our previous study has confirmed that 18G needle puncture can induce a progressive IVDD degeneration in rats weighed 400 g, while 26G needle can be used for intervertebral disc injection. In the current study, haematoxylin and eosin staining suggested that calcitonin was critical for sustaining the characteristics of chondrocytes and healthy NP tissues, and the normal intervertebral space height. Immunohistochemical staining showed that calcitonin treatment significantly increased the expression of type II collagen in the ECM of NPCs, further suggesting the vital role of calcitonin in the regulation of NPC ECM. Finally, immunohistochemical staining of PKC‐ε and PKC‐δ further confirmed the specific mechanism by which calcitonin regulates the NPC function: calcitonin delays the IVDD by activating PKC‐ε pathway while inhibiting PKC‐δ pathway. Although calcitonin cannot reverse intervertebral disc degeneration, it is effective in delaying degeneration process.

Although the present study reveals the mechanism by which calcitonin regulates the matrix components of NPCs, there are still some limitations. Further research is needed to identify PKC‐regulated downstream signals by which calcitonin exhibits its protective role in IVDD. Moreover, further studies will strengthen our understanding of the role of calcitonin on the molecular biology of IVDD as well as its potential application for developing therapeutic strategies for IVDD.

In summary, this study, for the first time, elucidates the important role of calcitonin in the regulation of matrix components in the nucleus of the intervertebral disc, and it clarifies the specific mechanism behind this effect. Calcitonin can reverse the cellular changes of the intervertebral disc nucleus by activating the PKC‐ε pathway and inhibiting the PKC‐δ pathway in vitro. These findings reveal new biological functions of calcitonin and provide a new theoretical basis for the specific mechanism of calcitonin prevention of IVDD, further deepening our understanding of the disease facilitating the development of new strategies for the prevention and treatment of IVDD. To our knowledge, this study reveals for the first time that calcitonin plays a critical role in the regulation of NP matrix of IVDD. Mechanistically, calcitonin activates PKC‐ε and inhibits PKC‐δ pathways to delay the degeneration of IVDD.

## COMPETING INTEREST STATEMENT

The authors declare no competing interests.

## AUTHOR CONTRIBUTION

Jun Ge: Formal analysis (equal); Investigation (lead); Resources (lead); Software (lead); Validation (lead); Visualization (lead); Writing‐original draft (lead); Writing‐review & editing (lead). Xiaoqiang Cheng: Formal analysis (equal); Investigation (equal); Methodology (lead); Resources (equal); Software (equal); Validation (equal); Visualization (equal); Writing‐original draft (equal); Writing‐review & editing (equal). Qi Yan: Resources (supporting); Software (supporting); Validation (supporting); Visualization (supporting); Writing‐original draft (supporting). Cenhao Wu: Resources (supporting); Software (supporting); Validation (supporting); Visualization (supporting); Writing‐review & editing (supporting). Yingjie Wang: Resources (supporting); Software (supporting); Writing‐review & editing (supporting). Hao Yu: Resources (supporting); Software (supporting); Visualization (supporting). Huilin Yang: Conceptualization (supporting); Data curation (supporting); Funding acquisition (supporting); Investigation (supporting); Methodology (supporting); Project administration (supporting). Feng Zhou: Conceptualization (equal); Data curation (lead); Formal analysis (equal); Investigation (lead); Methodology (lead); Project administration (lead); Supervision (lead); Validation (lead); Writing‐review & editing (equal). Jun Zou: Conceptualization (lead); Data curation (lead); Formal analysis (lead); Funding acquisition (lead); Investigation (lead); Methodology (lead); Project administration (lead); Supervision (lead); Validation (lead); Writing‐review & editing (lead).

## Data Availability

The data sets generated during and/or analysed during the current study are available from the corresponding author on reasonable request.
